# Electroacupuncture Attenuates Post-Inflammatory IBS-Associated Visceral and Somatic Hypersensitivity and Correlates With the Regulatory Mechanism of Epac1–Piezo2 Axis

**DOI:** 10.3389/fendo.2022.918652

**Published:** 2022-07-05

**Authors:** Jing Guo, Lu Chen, Yu-hang Wang, Ya-fang Song, Zhan-hao Zhao, Ting-ting Zhao, Zhi-ying Lin, Dong-mei Gu, Yun-qi Liu, Yong-jun Peng, Li-xia Pei, Jian-hua Sun

**Affiliations:** ^1^ Department of Acupuncture and Rehabilitation, The Affiliated Hospital of Nanjing University of Chinese Medicine, Nanjing, China; ^2^ Acupuncture and Massage College, Health and Rehabilitation College, Nanjing University of Chinese Medicine, Nanjing, China; ^3^ Department of Massage, Danyang Hospital of Traditional Chinese Medicine, Danyang, China; ^4^ Department of Acupuncture, Nantong Hospital of Traditional Chinese Medicine, Nantong, China; ^5^ Nanjing Foreign Language School, Nanjing, China

**Keywords:** electroacupuncture, irritable bowel syndrome, visceral hypersensitivity, somatic hypersensitivity, Epac1–Piezo2 axis

## Abstract

Electroacupuncture (EA) is considered to have a therapeutic effect in the relief of irritable bowel syndrome (IBS)-associated visceral hypersensitivity *via* the reduction of the level of 5‐hydroxytryptamine (5‐HT) and 5-HT_3_ receptors (5-HT_3_R). However, whether Epac1/Piezo2, as the upstream of 5-HT, is involved in this process remains unclear. We investigated whether EA at the ST36 and ST37 acupoints alleviated visceral and somatic hypersensitivity in a post-inflammatory IBS (PI-IBS) model mice *via* the Epac1-Piezo2 axis. In this study, we used 2,4,6-trinitrobenzene sulfonic acid (TNBS)-induced PI-IBS as a mouse model. Visceral sensitivity was assessed by the abdominal withdrawal reflex test. Somatic sensitivity was evaluated by the hind paw withdrawal threshold. Quantitative real-time PCR, immunofluorescence staining, ELISA, and Western blotting were performed to examine the expressions of Epac1, Piezo2, 5-HT, and 5-HT_3_R from the mouse distal colon/L5–S2 dorsal root ganglia (DRG). Our results showed that EA improved the increased visceral sensation and peripheral mechanical hyperalgesia in PI-IBS model mice, and the effects of EA were superior to the sham EA. EA significantly decreased the protein and mRNA levels of Epac1 and Piezo2, and reduced 5-HT and 5-HT_3_R expressions in the distal colon. Knockdown of colonic Piezo2 eliminated the effect of EA on somatic hypersensitivity. Combined knockdown of colonic Epac1 and Piezo2 synergized with EA in relieving visceral hypersensitivity and blocked the effect of EA on somatic hypersensitivity. Additionally, protein levels of Epac1 and Piezo2 were also found to be decreased in the L5–S2 DRGs after EA treatment. Taken together, our study suggested that EA at ST36 and ST37 can alleviate visceral and somatic hypersensitivity in PI-IBS model mice, which is closely related to the regulation of the Epac1–Piezo2 axis.

## Introduction

Irritable bowel syndrome (IBS) is a chronic functional bowel disorder characterized by abdominal pain associated with an alteration in bowel habits. IBS has a high prevalence and negatively affects patients’ social interactions and quality of life (1, 2). A subset of IBS, termed as post-inflammatory IBS (PI-IBS), occurs after an initial episode of acute gastrointestinal infection and exhibits persistent IBS-like symptoms. Despite the complex pathophysiology of IBS, visceral and somatic hypersensitivity may account for typical symptoms, such as urgent bowel movements, abdominal pain, or somatic symptoms (3, 4). There are several possible working models underlying this hypersensitivity, including nociceptive input from the colon, increased intestinal permeability, and inflammation in the colon (5).

The proliferation of enterochromaffin (EC) cells and increased release of 5‐hydroxytryptamine (5‐HT) have been shown to contribute to the development of visceral sensitization and peripheral mechanical hyperalgesia in IBS (6–9). It has been estimated that only 5% of the body’s 5-HT is stored in the central nervous system and 95% is stored in the gastrointestinal tract. In the latter, 90% of intestinal 5-HT is secreted by EC cells (10). EC cells are claimed to be specialized mechanosensory cells that release 5-HT in response to epithelial forces. Piezo2, known as a mechanically activated cation channel, is essential for the mechanosensitive EC cells and downstream physiological effects (11). Immunolabelling of Piezo2 revealed that Piezo2 has a specific distribution within EC cells of both human and mouse small intestinal epithelium (11). Activation of Piezo2 channels by force leads to inward currents in EC cells, which in turn lead to increased intracellular Ca^2+^, 5-HT release, and intestinal epithelial fluid secretion (12). It was found that Piezo1 and Piezo2 were expressed abundantly in intestinal epithelial cells of the colon. The expression of Piezo2 in the colon significantly correlated to visceral sensitivity in PI-IBS model mice, indicating Piezo2 might act as a candidate biomarker for visceral hypersensitivity in IBS (13). The Epac1 (exchange protein directly activated by cAMP1)–Piezo2 axis plays a role in the development of mechanical allodynia during neuropathic pain (14). Epac1 and Piezo2 expressions were significantly increased in the gastric fundic mucosa of visceral hypersensitivity model rats. *In vitro*, QGP-1 cells were used to mimic the mechanical stimuli felt by EC cells, and it was found that Epac1 participated in Piezo2-mediated mechanical signal transduction, which synergistically increased intracellular Ca^2+^ level and thus contributed to the 5-HT release (15).

Acupuncture is considered as a beneficial management of functional gastrointestinal disorders, including IBS (16). Several clinical studies have shown that acupuncture could alleviate abdominal pain in patients with IBS (17–19). Additionally, previous animal researches have reported the therapeutic effect of electroacupuncture (EA) treatment in the relief of IBS-associated visceral hypersensitivity and its effect on the reduction of the level of 5-HT and 5-HT3 receptor (5-HT_3_R) (20–23). However, whether Epac1/Piezo2 as the upstream of 5-HT is involved in this process remains unclear. EA at classic acupoints ST36 (Zusanli) and ST37 (Shangjuxu) has been proved to alleviate colorectal hypersensitivity (24). In this study, we investigated whether EA at ST36 and ST37 alleviated visceral and somatic hypersensitivity in 2,4,6-trinitrobenzene sulfonic acid (TNBS)-induced PI-IBS model mice *via* Epac1 and Piezo2. This may provide further understanding of EA treatment in PI-IBS.

## Materials and Methods

### Animals

Six-week-old C57BL/6J male mice weighing 16–18 g, which were purchased from Shanghai SLAC Laboratory Animal Co. Ltd (Shanghai, China), were housed under controlled conditions with an ambient temperature of 20–22°C, relative humidity of 40%–60%, a 12 h/12 h light/dark cycle, and ad libitum access to food and water. All animal care and experimental procedures complied with the Guidelines for the Care and Use of Laboratory Animals published by the United States National Institutes of Health and were approved by the Animal Ethics Committee in Affiliated Hospital of Nanjing University of Chinese Medicine (2021DW-24-01).

### Experimental Design

#### Experiment I

**Figure 1:**
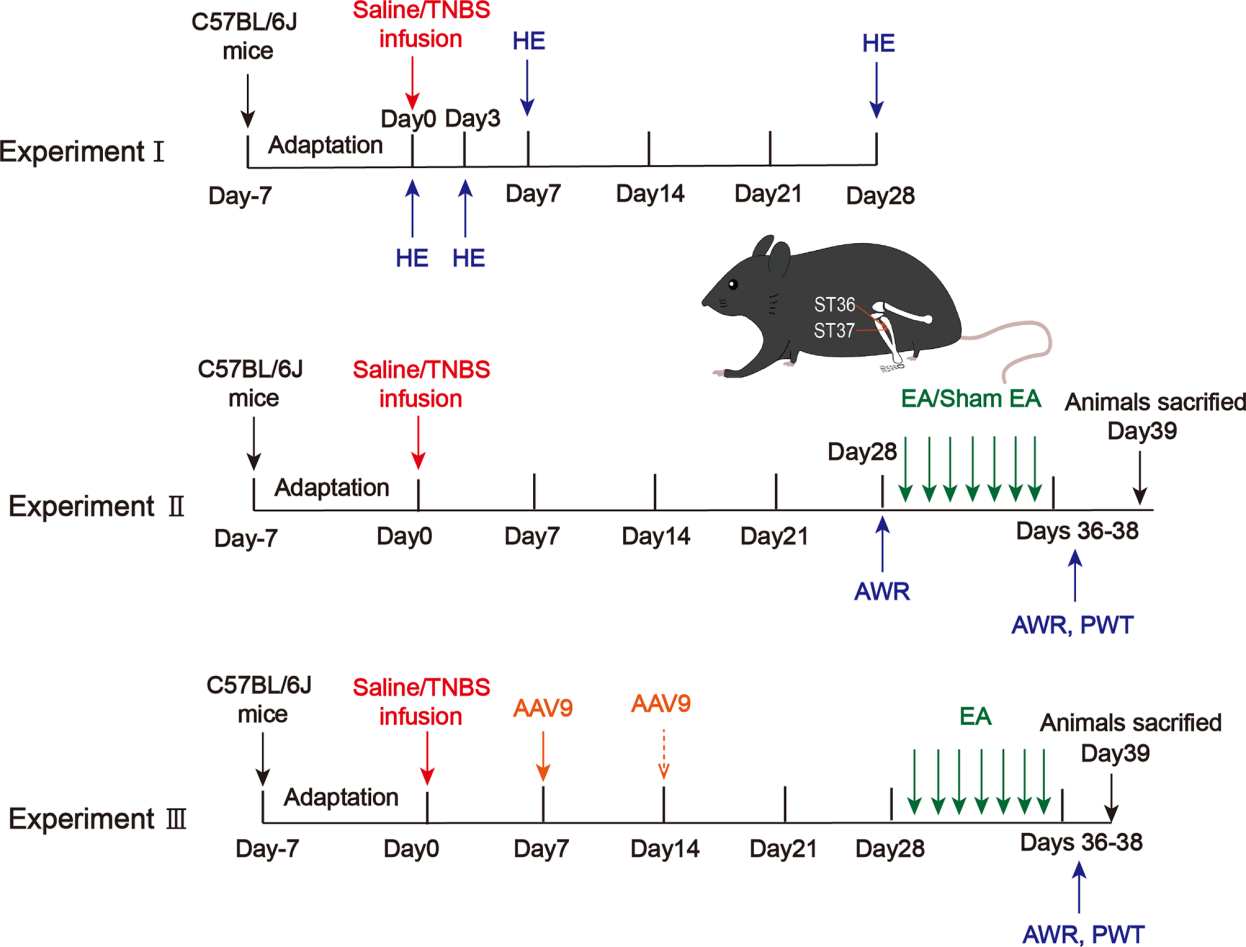
Protocols for electroacupuncture manipulation of the PI-IBS model mice. TNBS, 2,4,6-trinitrobenzene sulfonic acid; HE, hematoxylin and eosin of colonic tissues; AWR, abdominal withdrawal reflex; EA, electroacupuncture; Sham EA, sham electroacupuncture; ST36, acupoint Zusanli; ST37, acupoint Shangjuxu; PWT, paw withdrawal threshold.

To assess histological damage of colitis after TNBS infusion, mice were randomly assigned to 4 groups: day 0 (after saline intracolonic infusion), and 3, 7, 28 days after TNBS treatment (5 mice per group). After being carefully isolated, pathological changes in the distal colon were determined by Hematoxylin and Eosin Staining (HE) (**Figure 1**). Microscopic assessment was then blindly scored according to a semiquantitative scoring system with a total maximum score possible of 11 (**Supplementary Table 1**) (25, 26).

#### Experiment II

To determine the analgesic effect of EA on visceral and somatic hypersensitivity, mice were randomly assigned to 4 groups: control, model, EA, and sham EA (10 mice per group). Mice in the control group received an intracolonic infusion of 0.1 mL saline. Mice in the model, EA and sham EA groups received an intracolonic infusion of 0.1 mL TNBS (80mg/kg) in 50% ethanol to produce colitis (27). Four weeks post-TNBS administration, visceral pain threshold pressure was measured. Visceral sensitivity was assessed by the abdominal withdrawal reflex test (AWR), and somatic sensitivity was evaluated by the hind paw withdrawal threshold (PWT). The expressions of Epac1, Piezo2, 5-HT and 5-HT_3_R from the distal colon/L5–S2 DRGs were detected by quantitative real-time PCR, immunofluorescence staining, ELISA, and Western blotting (**Figure 1**).

#### Experiment III

To investigate whether colonic Epac1/Piezo2 is essential to the EA effect on visceral and somatic hypersensitivity, knockdown of Epac1/Piezo2 with AAV9 (shEpac1/shPiezo2) was used, and mice were randomly assigned to 8 groups: control, model, shRNA-control, shEpac1, shEpac1+EA, shPiezo2, shPiezo2+EA, EA. To investigate whether both colonic Epac1 and Piezo2 are essential to the EA effect on visceral and somatic hypersensitivity, mice were randomly assigned to 5 groups: control, model, shEpac1+shPiezo2, shEpac1+shPiezo2+EA, EA. AWR and PWT were measured. The expressions of Epac1/Piezo2 from the distal colon were detected by quantitative real-time PCR, and Western blotting. The expression levels of 5-HT were detected by ELISA (**Figure 1**).

### Mouse AAV9 Generation and Injection

The adeno-associated virus-9 (AAV9) vectors carrying Epac1 shRNA (shEpac1), Piezo2 shRNA (shPiezo2) and shRNA-control were constructed by Vigene Bioscience (Shangdong, China). The silence sequence targeting the Epac1 and Piezo2 genes are shown in the **Supplementary File**. AAV9 was administered *via* intraperitoneal injection with 1 × 10^12^ viral genome particles per mouse. Mice showed adequate transfection three weeks after AAV9 administration.

### Electroacupuncture Stimulation and Sham Electroacupuncture

Mice were anesthetized rapidly by 5% isoflurane inhalation *via* a precision vaporizer, maintained by 1.5% isoflurane with a mask, and laced on a heating pad to maintain body temperature during the intervention period. After disinfecting the skin with 75% alcohol, stainless steel acupuncture needles (0.19  × 10 mm, Hwato, China) were inserted 3 mm deep into the Zusanli (ST36) and Shangjuxu (ST37) acupoints. An electric current was provided to the needles by a Han’s Acupoint Nerve Stimulator (HANS-200A, Nanjing Gensun Medical Technology Co. Ltd., China) with parameters of 2/15 Hz, 1 mA for 15 min daily for seven consecutive days. Sham EA treatment included the same procedure but using a non-electrical wood “toothpick” through the pads without skin penetration instead of the electrodes (28).

### Abdominal Withdrawal Reflex

The AWR test was performed to measure visceral sensitivity. Mice were lightly anesthetized with isoflurane, and then an uninflated distension balloon (6-Fr, 2 mm in diameter) with paraffin oil was inserted into the distal colon. Mice were allowed to habituate in small plastic cubicles for 15 min. Graded distention was produced by inflating the balloon to a constant pressure (15, 30, 45, or 60 mmHg) for 20 s at 3 min intervals (24). The AWR scores (0–4, five scores) for each pressure were evaluated based on the AWR scoring system for each mouse in triplicate (**Supplementary Table 2**) (29).

### Hind Paw Withdrawal Threshold

The hind paw withdrawal threshold (PWT) was measured with a set of Von Frey hairs (North Coast, USA) with forces from 0.4 g to 15.0g. Von Frey measurements were done after the mice were placed in transparent plexiglass chambers on top of a raised wire mesh for 20 min and had adapted to the environment. A Von Frey filament starting at 2.0 g force was forced against the hind paw. We determined the 50% paw withdrawal threshold using the up-down method of Dixon and Chaplan’s formula (30, 31).

### Tissue Sample Collection

In Experiments II and III, at the end of the experiment (day 39), mice were humanely sacrificed with CO2 inhalation and cervical dislocation. The distal colon (length of 1 cm) and L5–S2 dorsal root ganglia (DRG) were quickly isolated, rinsed, and stored at -80°C or fixed in 4% paraformaldehyde solution. Freshly frozen tissues were then used for RNA extraction, protein fractionation, and ELISA. Fixed tissues were used for immunofluorescence staining and HE analysis.

### Hematoxylin and Eosin Staining

Hematoxylin and eosin staining was performed under standard histological protocols. After tissues fixed in 4% paraformaldehyde overnight, they were embedded in paraffin wax. Paraffin sections (4 μm) were cut with a manual rotary microtome (Leica, Germany) and stained with hematoxylin and eosin. Pathological changes in the samples visualized under a light microscope (Leica, Germany).

### Immunofluorescence Staining

The distal colon tissues and DRGs were fixed with 4% paraformaldehyde for 24 h, and cross-sections of the colonic tissues were obtained after being embedded in paraffin. The sections were stained with diaminobenzidine for 15 s. All sections were blocked with 5% goat serum and incubated with the primary antibody at 4°C overnight. The sections were then washed three times in phosphate-buffered saline (PBS) and incubated at room temperature with the secondary antibody for 1 h. For immunofluorescence analysis, the nuclei were counterstained with 4’,6‐diamino‐2‐phenylindole (DAPI). For primary antibodies, we used rabbit anti-Epac1 antibody (1:50, ab109415, Abcam, Cambridge, UK) or polyclonal rabbit anti−human Piezo2/FAM38B antibody (1:200, LS-C180179, LSBio, Seattle, USA) or serotonin antibody (1:200, sc-58031, Santacruz Biotech, Texas, USA). For secondary antibodies, we used goat anti-rabbit IgG H&L (HRP) (1:1000, ab205718, Abcam, Cambridge, UK), and goat anti-rabbit IgG H&L (Alexa Fluor^®^ 488) (1:1000, ab150077, Abcam, Cambridge, UK). The samples were then viewed under a fluorescence microscope (Olympus, Japan).

### Quantitative Real-Time PCR

Total RNA was extracted from tissue biopsies by Trizol (Jialan Biology, Beijing, China). Complementary DNA was synthesized using the RT Regent Kit with gDNA Eraser (Perfect Real Time; TaKara, Dalian, China). Quantitative real-time PCR (qPCR) was carried out using the Hieff^®^ qPCR SYBR Green Master Mix (High Rox Plus) (11203ES03, YEASEN, Shanghai, China) on the StepOnePlus Real-Time PCR System (Applied Biosystems, CA, USA). *GAPDH* was used as internal reference to normalize the expression levels. The relative expression was calculated using the 2^−ΔΔCt^ method. Method of primer design and primer sequences are shown in the **Supplementary Table 3**.

### Western Blotting

The colonic tissues and DRGs were homogenized with RIPA lysis buffer. After cooling in an ice-water bath, the tissues were immediately centrifuged at 10000 rpm and 4°C for 10 minutes, and the supernatant was removed for testing. Enhanced BCA Protein Assay Kit (P0010) was used to measure protein concentrations. Equal amounts of protein were separated by SDS-PAGE and transferred to PVDF membranes. The membranes were blocked with 5% non-fat milk for 1 h at room temperature before being probed with rabbit anti-Epac1 antibody (1:1000, ab109415, Abcam, Cambridge, UK) or polyclonal rabbit anti−human Piezo2/FAM38B antibody (1:1000, LS-C180179, LSBio, Seattle, US) or rabbit anti-HTR3A antibody (1:500, 10443-1-AP, Proteintech, Chicago, USA) at 4°C overnight. After washing the membranes, they were subsequently incubated with goat anti-rabbit IgG (H+L) HRP (1:5000, S0001, Affinity Biosciences, Jiangsu, China) at room temperature for 1 h. Protein expression was quantified using Image J (National Institutes of Health, Bethesda, Maryland, USA).

### ELISA

Colon tissues o were homogenized in saline and supernatants were extracted by centrifugation at 3000 rpm for 10 min at 4°C. 5-HT was determined using a mouse 5-HT ELISA kit (ZC-37715, ZCIBIO, Shanghai, China) according to the manufacturer’s instructions. Standard or sample solutions (50 µl) were added to the 96 wells. Then, horseradish peroxidase-labeled 5-HT antibody (100 µl) was added to the wells and incubation was performed at 37°C for 60 min. Optical density values were measured at a wave length of 450 nm.

### Statistical Analysis

All statistical analyses were conducted using SPSS 23.0 (SPSS, Chicago, USA). Original image productions were performed using Prism 7.0 software (Prism, CA, USA). Data are presented as the mean ± standard error of the mean (SEM). Comparisons between the two groups were assessed using two-tailed *t*-test or Mann–Whitney test. Data among more than two groups were compared using one-way analysis of variance followed by LSD *post-hoc* test. Data from body weight were analyzed using generalized estimating equations (GEE) due to the correlated structure of data from repeated measures at different time points. A two-sided *P* value of < 0.05 was considered statistically significant.

## Results

### EA Attenuated Visceral and Somatic Hypersensitivity in TNBS-Induced PI-IBS Model Mice

**Figure 2:**
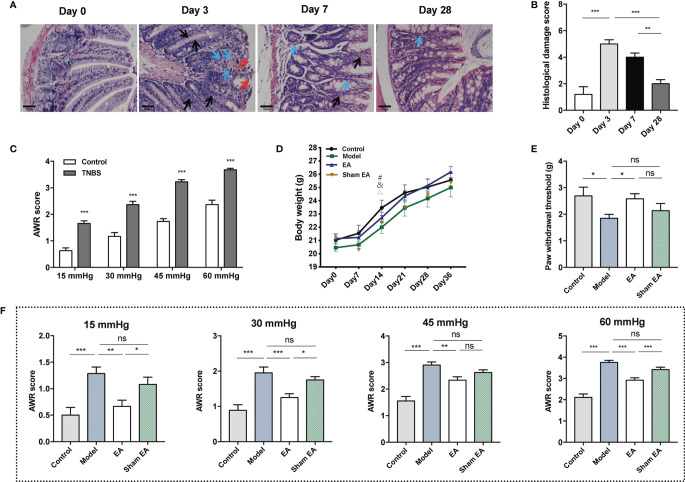
Effect of EA on visceral and somatic hypersensitivity in PI-IBS model mice. **(A)** Representative HE images of the colon tissues in TNBS treated mice at day 0 (after saline intracolonic infusion), and 3, 7, 28 days after TNBS treatment (scale bar = 50 μm). Red arrowheads show destruction of mucosal architecture. Blue arrowheads represent cellular infiltration. Black arrowheads show goblet cell depletion. **(B)** Histological damage scoring of the colon tissues (n=5). **(C)** Abdominal withdrawal reflex scores indicating at stimulation pressures of 15, 30, 45, and 60 mmHg in control (n = 10) and TNBS treated mice (n = 30). **(D)** Body weight of mice during the experiment I. **(E)** Hind paw withdrawal threshold evaluated after the treatment of electroacupuncture/sham electroacupuncture (n = 6 per group). **(F)** Abdominal withdrawal reflex (AWR) scores at stimulation pressures of 15, 30, 45, and 60 mmHg in each group (n = 6 per group). Data are presented as mean ± SEM; **(B, E**, and **F)**: One-way ANOVA with LSD multiple comparisons test; **(C)**: two-tailed Mann–Whitney test; **(D)**: generalized estimating equations, ^#^: model versus control, ^&^: EA versus control, ^△^: sham EA versus control, *P* < 0.05; ^*^ *P* < 0.05, ^**^ *P* < 0.01, ^***^ *P* < 0.001, ^ns^ *P*>0.05.

Compared to mice at day 0, significant damage was present in the acute inflammatory mice (days 3 and 7). On day 3, the absence of crypt abscesses and goblet cells, and moderate destruction of mucosal architecture and cellular infiltration were observed. The figure on day 7 showed that the absence of goblet cells, mild to moderate cellular infiltration, and mild muscle thickening (**Figure 2A**). Histological examination of TNBS treated colons demonstrated significant damage was induced after three days of TNBS treatment and was negligible by day 28, indicating inflammatory recovery (**Figure 2B**). The TNBS treated mice at day 28 had a higher AWR score than that of mice in the control group under 15 mmHg, 30 mmHg, 45 mmHg, and 60 mmHg pressure stimulation (**Figure 2C**). These results indicate the successful induction of TNBS induced PI-IBS model in the mice.

After seven days of TNBS infusion, body weight decreased significantly in the sham EA group. In the control, model and EA groups, body weight tended to increase, while the difference was not significant. At day 14, body weight in the model, EA, and sham EA groups were lower than in the control. At other time points, there was no significant difference in body weight between the four groups (**Figure 2D**; **Supplementary Table 3**). Compared with the control group, lower PWT was observed in the model group. EA treatment increased PWT significantly compared to the model group (**Figure 2E**). The AWR scores of the model group were significantly higher than that of the control group under four different levels of pressure (**Figure 2F**). The EA group had lower AWR scores than the model group (**Figure 2F**). The AWR scores of the EA group were lower than those of the sham EA group under 15 mmHg, 30 mmHg, and 60 mmHg pressure (**Figure 2F**). Taken together, EA can decrease the mechanic or nociceptive sensitivity of the PI-IBS mice.

### EA Decreased the Expressions of Epac1, Piezo2, 5-HT, and 5-HT_3_R in the Distal Colonic Tissues

**Figure 3:**
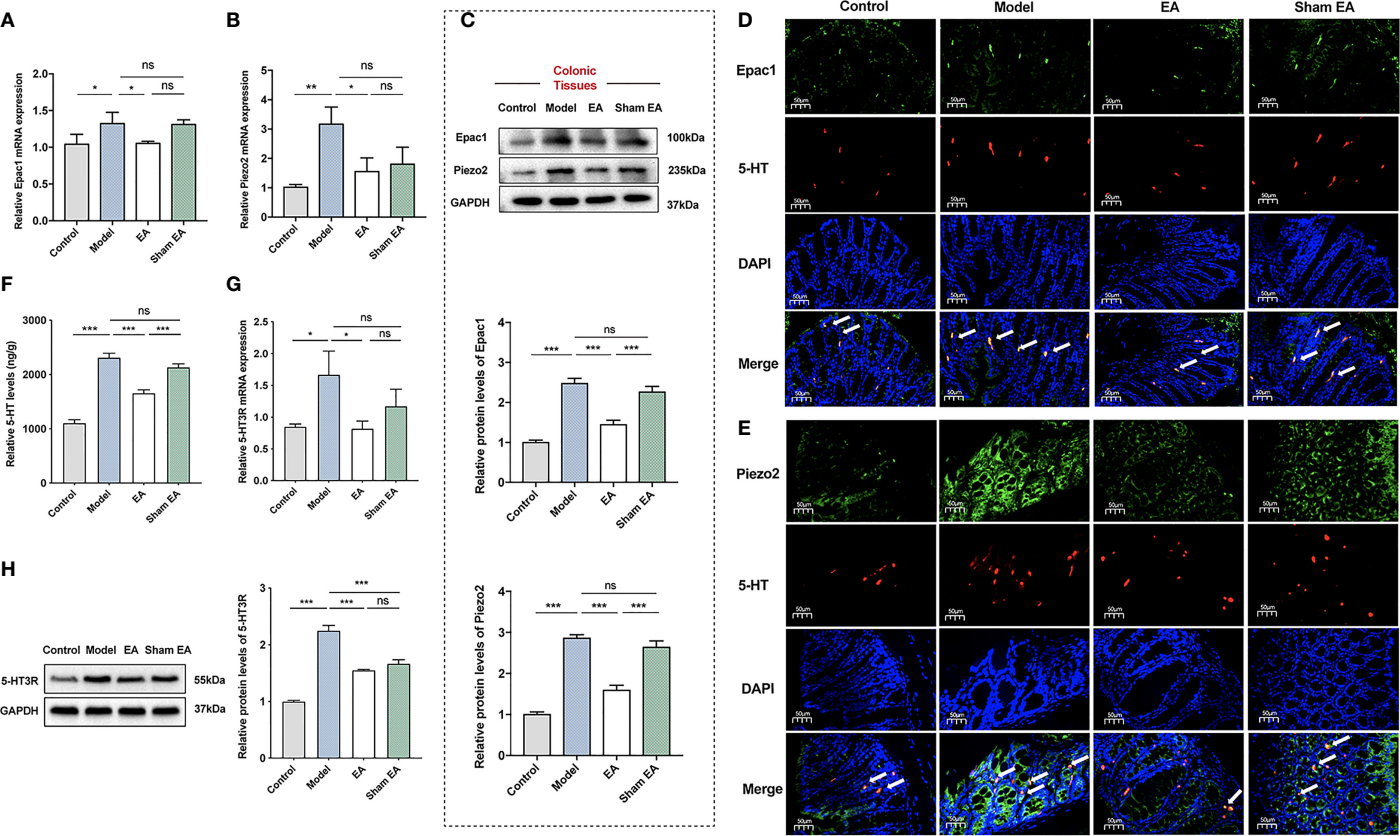
Effect of EA on the expression of Epac1, Piezo2, 5-HT, and 5-HT_3_R in the colon of PI-IBS model mice. **(A)** Relative colonic Epac1 mRNA expression (n=5). **(B)** Relative colonic Piezo2 mRNA expression (n=5). **(C)** Representative Western blot analyses of Epac1 and Piezo2 staining and relative protein levels in the colonic tissues (n=5). **(D, E)** Representative images showing Epac1/Piezo2 (green), 5-HT (red), and DAPI (blue) fluorescence in different groups (×400, scale bar=50 μm). The arrow head in the merged image indicate co-expression (n=3). **(F)** ELISA analysis of 5-HT in colonic tissues (n=6). **(G)** Relative colonic 5-HT_3_R mRNA expression (n=5). **(H)** Representative Western blot analyses of 5-HT_3_R staining and relative protein levels in the colonic tissues (n=3). Data are presented as mean ± SEM; **(A-C, F-H)**: One-way ANOVA with LSD multiple comparisons test; ^*^ *P* < 0.05, ^**^ *P* < 0.01, ^***^ *P* < 0.001, ^ns^ *P*>0.05.

We first used qPCR, Western blotting, and immunofluorescence staining to assess the expression of Epac1 and Piezo2 in the distal colonic tissues. Colonic Epac1 mRNA expression level was higher in the model group than in the control group (**Figure 3A**). Compared with the model group, the expression of Epac1 mRNA in the colonic tissues was significantly reduced by EA stimulation (**Figure 3A**). Increased colonic Piezo2 mRNA expression was observed in the model group compared with the control group and EA could reduce the Piezo2 mRNA expression (**Figure 3B**). Similar results were also found for Epac1 and Piezo2 by immunofluorescence staining and Western blotting (**Figures 3C–E**). Additionally, the EA group expressed lower colonic Epac1 and Piezo2 protein levels than the sham EA group (**Figures 3C–E**).

5-HT is a neurotransmitter in the gut, which has many effects related to IBS, including stimulating intestinal secretion and colonic motility (32). It has been reported that 5‐HT_3_ receptor (5‐HT_3_R) antagonists, which could block these effects, are effective treatments for IBS‐D (33, 34). To investigate the effect of EA on the expression of 5-HT and its main receptor, we measured the expression of 5-HT and 5-HT_3_R in the colon. The model group had a higher 5-HT level than the control group (**Figure 3F**). EA at ST36 and ST37 decreased the colonic 5-HT level in comparison with that in PI-IBS model mice. The 5-HT of the EA group was much lower than that of the sham EA group. The expression levels of 5-HT_3_R mRNA and 5-HT_3_R of the colon in the model group were significantly increased compared with the control group (**Figures 3G, H**), and EA treatment reduced the expressions of 5-HT_3_R mRNA and 5-HT_3_R.

### EA Reduced the Expressions of Epac1 and Piezo2 in the L5–S2 DRGs

**Figure 4:**
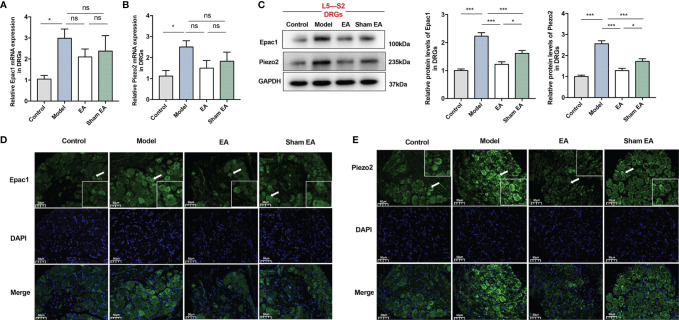
Effect of EA on the expression of Epac1 and Piezo2 in the L5-S2 dorsal root ganglia of PI-IBS model mice. **(A)** Relative Epac1 mRNA and **(B)** Piezo2 mRNA expressions in the dorsal root ganglia. **(C)** Representative Western blot analyses of Epac1 and Piezo2 staining and relative protein levels in the dorsal root ganglia. **(D, E)** Representative images of immunofluorescence labeling of Epac1 and Piezo2 (×400, scale bar=50 μm). Arrowheads indicate Epac1 or Piezo2 positive cells. Boxes represent the local magnification of positive cells. Data are presented as mean ± SEM; **(A-C)**: One-way ANOVA with LSD multiple comparisons test; n = 5 per group, ^*^ *P* < 0.05, ^***^ *P* < 0.001, ^ns^ *P*>0.05.

The DRG is a critical structure in sensory transduction and modulation, including pain transmission (35). For further investigations, we also observed Epac1 and Piezo2 expressions in the L5–S2 DRGs. The expression levels of Epac1 mRNA and Piezo2 mRNA of the DRGs in the model group were significantly increased compared with the control group (**Figures 4A, B**). As is shown in **Figures 4A, B**, EA treatment tended to decrease mRNA expression levels of Epac1 and Piezo2, but there was no statistically significant difference. There was no difference in Epac1 and Piezo2 mRNA between the model group and sham EA group. Epac1 and Piezo2 expressions in the model group were significantly increased compared with the control group (**Figures 4C–E**), and EA treatment significantly reduced Epac1 and Piezo2 expressions (**Figures 4C–E**). Epac1 and Piezo2 expressions in the DRGs were lower in the EA group than in the sham EA group (**Figures 4C–E**).

### Knockdown of Colonic Epac1 Didn’t Influence the Effect of EA on Visceral and Somatic Hypersensitivity

**Figure 5:**
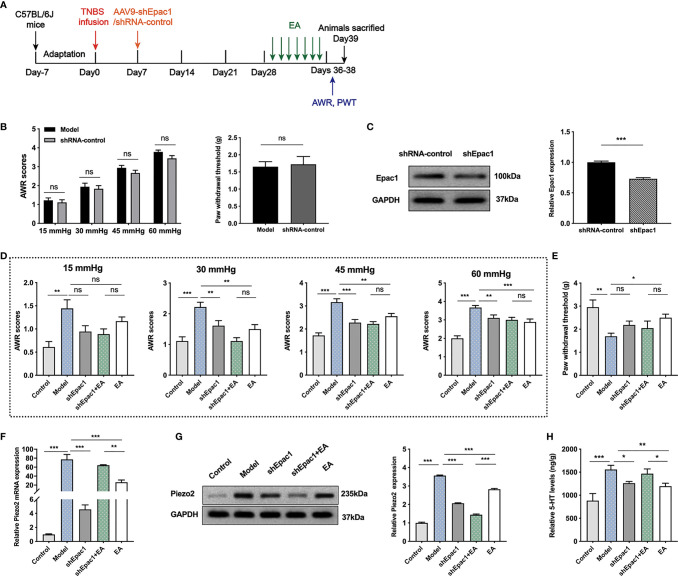
Knockdown of colonic Epac1 didn’t influence the effect of EA. **(A)** Schedule of the experimental procedures. **(B)** Abdominal withdrawal reflex and hind paw withdrawal threshold evaluated between the model and shRNA-control groups (n=6 per group). **(C)** Representative Western blot analyses of Epac1 staining and relative protein levels between the shRNA-control and shEpac1 groups (n=3 per group). **(D, E)** Abdominal withdrawal reflex scores and hind paw withdrawal threshold evaluated in each group (n=6 per group). **(F)** Relative colonic Piezo2 mRNA expression (n=3 per group). **(G)** Representative Western blot analyses of Piezo2 staining and relative protein levels in the colonic tissues (n=3 per group). **(H)** ELISA analysis of 5-HT in colonic tissues (n=6 per group). Data are presented as mean ± SEM; **(B)** two-tailed Mann–Whitney test for AWR, *t* test for PWT; **(C)** two-tailed *t* test; **(D-H)** One-way ANOVA with LSD multiple comparisons test; ^*^ *P* < 0.05, ^**^ *P* < 0.01, ^***^ *P* < 0.001, ^ns^ *P*>0.05.

To further investigate whether colonic Epac1 is essential to the EA effect on visceral and somatic hypersensitivity, we inhibited Epac1 in colonic tissues with the AAV9 vector (**Figure 5A**). The AWR scores and PWT in the shRNA-control group were similar to the model group, indicating that intraperitoneal injection of AAV9-shRNA-control did not affect the visceral and somatic hypersensitivity of the PI-IBS model mice (**Figure 5B**). Injection of AAV9-shEpac1 could successfully inhibit Epac1 expression in the colonic tissues of the PI-IBS model mice (**Figure 5C**). Compared with the model group, AWR scores in the shEpac1 and EA groups were lower under 30, 45, 60 mmHg (**Figure 5D**). EA, not shEpac1, could increase PWT (**Figure 5E**). There were no significant differences in AWR scores and PWT between the shEpac1+EA group and EA group (**Figures 5D, E**). EA and injection of shEpac1 could decrease Piezo2 mRNA, Piezo2, and 5-HT in the colonic tissues (**Figures 5F–H**). Piezo2 mRNA expression in the shEpac1+EA group was higher than the EA group, while the protein expression level showed an opposite trend (**Figures 5F, G**). The shEpac1+EA group had higher 5-HT levels than the EA group (**Figure 5H**).

### Knockdown of Colonic Piezo2 Eliminated the Effect of EA on Somatic Hypersensitivity

**Figure 6:**
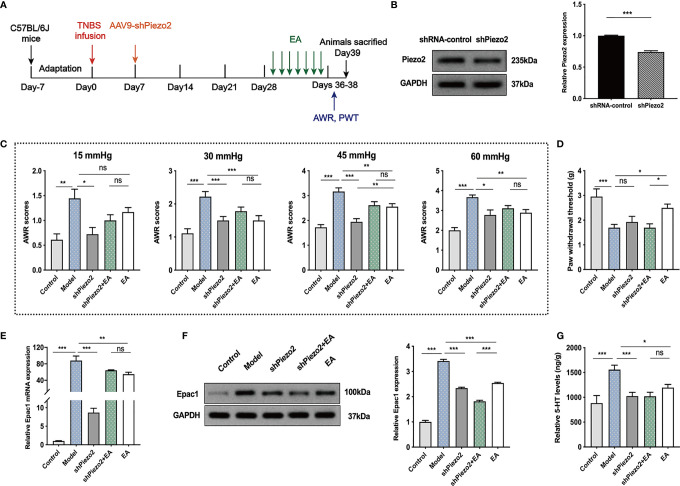
Knockdown of Piezo2 eliminated the effect of EA on somatic hypersensitivity. **(A)** Schedule of the experimental procedures. **(B)** Representative Western blot analyses of Piezo2 staining and relative protein levels between the shRNA-control and shPiezo2 groups (n=3 per group). **(C, D)** Abdominal withdrawal reflex scores and hind paw withdrawal threshold evaluated in each group (n=6 per group). **(E)** Relative colonic Epac1 mRNA expression (n=3 per group). **(F)** Representative Western blot analyses of Epac1 staining and relative protein levels in the colonic tissues (n=3 per group). **(G)** ELISA analysis of 5-HT in colonic tissues (n=6 per group). Data are presented as mean ± SEM; **(B)** two-tailed *t* test; **(C-G)** One-way ANOVA with LSD multiple comparisons test; ^*^ *P* < 0.05, ^**^ *P* < 0.01, ^***^ *P* < 0.001, ^ns^ *P*>0.05.

To further investigate whether colonic Piezo2 is essential to the EA effect on visceral and somatic hypersensitivity, we inhibited Piezo2 in colonic tissues (**Figure 6A**). Injection of AAV9-shPiezo2 could successfully inhibit Piezo2 expression in the colonic tissues (**Figure 6B**). AWR scores in shPiezo2 and EA groups were lower than the model group (**Figure 6C**). There were no significant differences in AWR scores between the shPiezo2+EA group and EA group (**Figure 6C**). EA could increase PWT, and PWT in the shPiezo2+EA group was lower than EA group (**Figure 6D**). EA and injection of shPiezo2 could decrease Epac1 mRNA, Epac1, and 5-HT in the colonic tissues (**Figures 6E–G**). The protein expression level of Epac1 expression in the shPiezo2+EA group was lower than in the EA group (**Figure 6F**). 5-HT expression levels in colonic tissues were similar in the shPiezo2+EA and EA groups (**Figures 6G**).

### Combined Knockdown of Colonic Epac1 and Piezo2 Synergized With EA in Relieving Visceral Hypersensitivity and Eliminated the Effect of EA on Somatic Hypersensitivity

**Figure 7:**
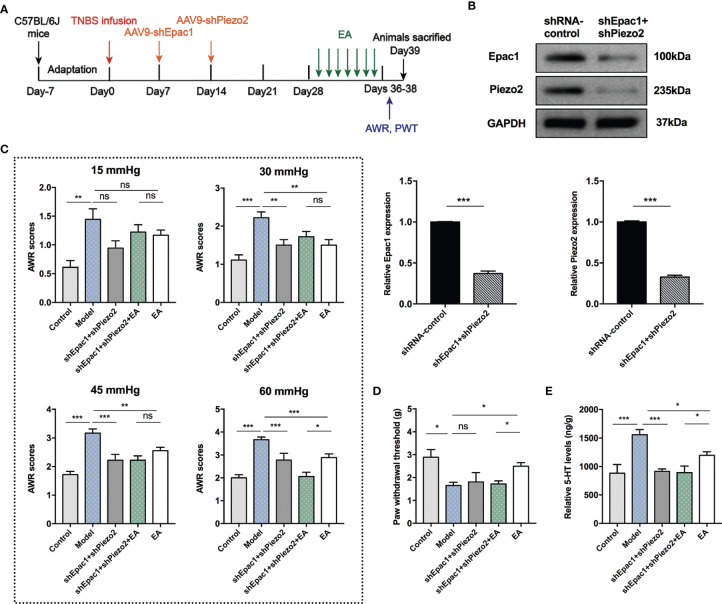
Combined knockdown of colonic Epac1 and Piezo2 synergized with EA in relieving visceral hypersensitivity and eliminated the effect of EA on somatic hypersensitivity. **(A)** Schedule of the experimental procedures. **(B)** Representative Western blot analyses of Epac1 and Piezo2 staining and relative protein levels between the shRNA-control and shEpac1+shPiezo2 groups (n=3 per group). **(C, D)** Abdominal withdrawal reflex scores and hind paw withdrawal threshold evaluated in each group (n=6 per group). **(E)** ELISA analysis of 5-HT in colonic tissues (n=6 per group). Data are presented as mean ± SEM; **(B)** two-tailed *t* test; **(C-E)** One-way ANOVA with LSD multiple comparisons test; ^*^ *P* < 0.05, ^**^ *P* < 0.01, ^***^ *P* < 0.001, ^ns^ *P*>0.05.

Knockdown of Epac1 or Piezo2 in colonic tissues did not eliminate the effect of EA in relieving visceral hypersensitivity in PI-IBS model mice. Therefore, we further investigated whether a combined knockdown of colonic Epac1 and Piezo2 would affect the effect of EA on visceral and somatic hypersensitivity (**Figure 7A**). Combined injection of AAV9-shEpac1 and AAV9-shPiezo2 could successfully inhibit Epac1 and Piezo2 expressions in the colonic tissues (**Figure 7B**). Compared with the model group, AWR scores in the shEpac1+shPiezo2 and EA groups were lower under 30, 45, 60 mmHg (**Figure 7C**). The AWR scores in the shEpac1+shPiezo2+EA group were lower than those in the EA group under 60 mmHg (**Figure 7C**). EA could increase the PWT in PI-IBS model mice, and PWT in the shEpac1+shPiezo2+EA group was lower than the EA group (**Figure 7D**). Both shEpac1+shPiezo2 and EA could reduce 5-HT levels in the colonic tissues, and the shEpac1+shPiezo2+EA group had lower 5-HT levels than the EA group (**Figure 7E**).

## Discussion

In this study, we have presented evidence demonstrating the involvement of increased Epac1 and Piezo2 expression levels in the pathogenesis of visceral sensitization and peripheral mechanical hyperalgesia in TNBS-induced PI-IBS model mice. EA at ST36 and ST37 acupoints could ameliorate visceral and somatic hypersensitivity by reducing Epac1 and Piezo2 in the colon, which decreased 5-HT expression. Additionally, down-regulation of Epac1 and Piezo2 by EA in the L5–S2 DRGs were also observed. Knockdown of colonic Piezo2 eliminated the effect of EA on somatic hypersensitivity. Combined knockdown of colonic Epac1 and Piezo2 synergized with EA in relieving visceral hypersensitivity and blocked the effect of EA on somatic hypersensitivity. To our knowledge, this is the first study focusing on the Epac1–Piezo2 axis to explore the possible mechanism of EA for visceral and somatic hypersensitivity in PI-IBS.

In 2010, Ardem Patapoutian and his colleagues used a functional screen of candidate genes expressed in a mouse neuroblastoma cell line to identify ion channels activated by mechanical stimuli. Among the 72nd candidate genes, they found that *FAM38A* functions as a mechanical sensor, which was then named Piezo1. Later on, based on sequence homology comparison, they found *FAM38B* (Piezo2) (36). Meanwhile, Patapoutian also demonstrated that Piezo2 is the major mechanical transducer in cutaneous mechanoreceptors and a subset of sensory neurons of the DRG, and is required for our perception of touch and proprioception (37). Strikingly, Patapoutian was awarded the 2021 Nobel Prize in Physiology or Medicine for his discovery of the mechanisms related to mechanic perception (38). Our previous bibliometric analysis shows that the research focus of the Piezo channel has evolved from Piezo identification to architecture, activation mechanism, pharmacology, and roles in diseases (39).

Piezo2 is expressed in gastrointestinal epithelial EC cells and gut-associated DRGs, and is involved in gastrointestinal sensory regulation. Interestingly, Bai T et al. found that the expression level of Piezo2 in the colon is higher than that in the small intestine (13). Piezo2 is important in the gastrointestinal tract where the EC cells are inherently mechanosensitive and release 5-HT in response to mechanical force from gastrointestinal luminal content (11, 12). It has been indicated that the proliferation of EC cells and increased 5‐HT expression are involved in visceral hypersensitivity of IBS (6–9). It was hypothesized that increased expression of Piezo2 could influence visceral sensitivity *via* modulation of 5-HT release. Bai T et al. provided the evidence that expression of Piezo2 in the colon is significantly correlated with the visceral sensitivity in PI-IBS model mice induced *via* a *Trichinella spiralis* infection (13). The Epac1–Piezo2 axis has been reported to participate in EC cells releasing 5-HT. Epac1 and Piezo2 were significantly increased in the gastric fundic mucosa of visceral hypersensitivity model rats (15). Our results also presented increased Epac1 and Piezo2 expressions, and 5-HT concentration in the colon of PI-IBS model mice. Knockdown of colonic Epac1 or Piezo2 alleviated visceral hypersensitivity in PI-IBS model mice.

The analgesic effect of EA on visceral hypersensitivity has been extensively demonstrated (40–44), and the results of our study are consistent with this. EA reduced AWR scores and increased PWT, indicating that EA attenuated the visceral sensitization and peripheral mechanical hyperalgesia of PI-IBS. Sham EA showed a slight improvement in visceral and somatic hypersensitivity, and the analgesic effect of EA was superior to sham EA. Multiple studies suggest that EA alleviates visceral hypersensitivity *via* reduction of colonic EC cell number, 5-HT concentration and 5-HT_3_R expression (20–23). As has been found in previous research, decreased 5-HT concentration, and 5-HT_3_R expression in the EA group were also observed in our study. Having identified that the effects of EA on visceral hypersensitivity are closely related to 5-HT and its receptors, we wonder whether the Epac1–Piezo2 axis, which is known as a factor in the process of EC cells releasing 5-HT, is involved in the effect of EA on visceral hypersensitivity. The result showed the colonic Epac1 and Piezo2 expressions in the EA group were significantly lower than those in the model group. Colonic Piezo2 mediated the effect of EA in alleviating somatic hypersensitivity. Combined knockdown of colonic Epac1 and Piezo2 synergized with EA in relieving visceral hypersensitivity and abolished the effect of EA on somatic hypersensitivity.

The high expression of Piezo2 in the DRG and its co-localization with small-diameter unmyelinated nociceptors in the DRG suggests that it may be involved in the distal colorectal response to noxious mechanical stimuli (45). Intrathecal injection of Piezo2-short hairpin RNA (shRNA) decreased Piezo2 expression in the lumbosacral DRG. Piezo2 knock-down in DRG attenuated the response to innocuous stimuli in control rats and to innocuous and noxious stimuli in rats with neonatal irritation, suggesting the role of Piezo2 in visceral sensation (46). The Epac1–Piezo2 axis has a role in the development of mechanical allodynia (14, 47). Meanwhile, one study reported that Epac1 might be a potential target for the treatment of visceral hypersensitivity. Epac1 protein and mRNA expressions were elevated in the DRGs in a rat model of visceral hypersensitivity, and intrathecal injection of Epac1 inhibitor improved visceral hypersensitivity (48). In our study, we also found that Epac1 and its mRNA were increased in the L5–S2 DRGs of TNBS-induced PI-IBS mice. In addition, expressions of Piezo2 protein and mRNA in the model group were higher than those of the control group.

DRG, as a cytosol for primary afferent neurons, occupies a unique position within the nociceptive pathway. Sensitization of visceral DRG neurons could contribute to triggering visceral hypersensitivity (49). It is reported that DRG plays an important role in the regulation of visceral hypersensitivity in IBS by EA (41, 50, 51). EA treatment can significantly inhibit the enhanced excitability of colon DRG neurons and reduce the peripheral sensitization status (41, 50). We found that EA at ST36 and ST37 can reduce Epac1 and Piezo2 expressions in the L5–S2 DRGs of PI-IBS model mice and alleviate visceral hypersensitivity. This provides a new perspective to explore the mechanisms of EA for visceral hypersensitivity based on the DRG level.

There are several limitations in this research. We found that EA at ST36 and ST37 attenuated PI-IBS associated visceral and somatic hypersensitivity, and correlated with the regulatory mechanism of Epac1 and Piezo2 both in the colon and L5–S2 DRGs. How inhibition of colonic Epac1 or Piezo2 influences their levels in the DRG neurons has not been investigated. Moreover, the regulatory mechanism of EA in the decrease of Epac1 and Piezo2 in the DRGs has not been clarified. For the next step, we will use AAV9 vectors carrying shEpac1 or shPiezo2 for knockdown of Epac1 or Piezo2 in the DRGs to explore whether this will eliminate the analgesic effect of EA. Different results of inhibition of colonic Epac1 and Piezo2 influencing EA's effect on visceral and somatic hypersensitivity differently need to be interpreted in the future.

In summary, in the present study, we demonstrate that EA at ST36 and ST37can alleviate visceral and somatic hypersensitivity in TNBS-induced PI-IBS model mice, which is probably related to the decreased expression of Epac1 and Piezo2 both in the colon and L5–S2 DRGs, and the reduced 5-HT release and 5-HT_3_R expression. Colonic Piezo2 mediated the effect of EA in alleviating somatic hypersensitivity.

## Data Availability Statement

The original contributions presented in the study are included in the article/**Supplementary Material**. Further inquiries can be directed to the corresponding authors.

## Ethics Statement

The animal study was reviewed and approved by Animal Ethics Committee in Affiliated Hospital of Nanjing University of Chinese Medicine.

## Author Contributions

JG, L-XP conceived the study; J-HS and Y-JP supervised the study; JG, YF-S, T-TZ, Z-YL, and D-MG performed the experiments; JG, Y-HW and Z-HZ analyzed data; JG, LC, J-HS contributed to the discussion of the findings; JG and L-XP drafted the manuscript; J-HS, Y-JP, JG and Y-QL revised the manuscript; and all authors approved the final manuscript.

## Funding

This study was supported by the Jiangsu Postgraduate Practice Innovation Program (grant no. SJCX21_0737); National Natural Science Foundation of China (grant no. 81804193); Social Development Special Project of Jiangsu Science and Technology Department (grant no. BE2020788); National Administration of Traditional Chinese Medicine (grant no. GZY-KJS-2020-07).

## Conflict of Interest

The authors declare that the research was conducted in the absence of any commercial or financial relationships that could be construed as a potential conflict of interest.

## Publisher’s Note

All claims expressed in this article are solely those of the authors and do not necessarily represent those of their affiliated organizations, or those of the publisher, the editors and the reviewers. Any product that may be evaluated in this article, or claim that may be made by its manufacturer, is not guaranteed or endorsed by the publisher.

## Abbreviations

EA, Electroacupuncture; IBS, irritable bowel syndrome; PI-IBS, post-inflammatory irritable bowel syndrome; 5‐HT, 5‐hydroxytryptamine; DRG, dorsal root ganglia; Epac1, exchange protein directly activated by cAMP1; AWR, abdominal withdrawal reflex; PWT, paw withdrawal threshold
