# Supplementary material for: Electroacupuncture Attenuates Post-Inflammatory IBS-Associated Visceral and Somatic Hypersensitivity and Correlates With the Regulatory Mechanism of Epac1–Piezo2 Axis
Source: Front Endocrinol (Lausanne). 2022 Jul 5;13:918652. doi: 10.3389/fendo.2022.918652 (PMC9294163; doi:10.3389/fendo.2022.918652)
Supplement: Supplementary file 1 [file DataSheet_1.docx]

**Supplementary Content**

- Table 1. Histological scoring based on a semiquantitative scoring system
- The silence sequence targeting the Epac1 and Piezo2 genes for mouse AAV9-shEpac1 and AAV9-shPiezo2 generation
- Table 2. Abdominal withdrawal reflex (AWR) scoring criteria.
- Table 3. Method of primer design and primer sequences for quantitative real-time PCR
- Table 4. Body weight of mice during the experiment Ⅱ.

**Table 1. Histological scoring based on a semiquantitative scoring system** with a total maximum score possible of 11, including extent of destruction of normal mucosal architecture, presence and degree of cellular infiltration, extent of muscle thickening, presence or absence of crypt abscesses and the presence or absence of goblet cell depletion.

| **Score** | **destruction of mucosal architecture** | **presence and**  **degree of cellular infiltration** | **muscle thickening** | **crypt abscesses** | **goblet**  **cell depletion** |
| --- | --- | --- | --- | --- | --- |
| 0 | normal | normal | normal | absent | absent |
| 1 | mild | mild | mild | present | present |
| 2 | moderate | moderate | moderate |  |  |
| 3 | extensive damage | transmural infiltration | extensive thickening |  |  |

**The silence sequence targeting the Epac1 and Piezo2 genes for mouse AAV9-shEpac1 and AAV9-shPiezo2 generation**

- **The silence sequence targeting the Epac1 gene is as follows:**

Epac1-sh1：GCACCTACATCTGCAACAAGATTCAAGAGATCTTGTTGCAGATGTAGGTGCTTTTTT

Epac1-sh2：GGTGGAGCTGATCCATTATTTCAAGAGAATAATGGATCAGCTCCACCTTTTTT

Epac1-sh3：GGTGCCCTTTGTCATGTAATTCAAGAGATTACATGACAAAGGGCACCTTTTTT

Epac1-sh4：GCTACTCAGGAAGTTCATCAATTCAAGAGATTGATGAACTTCCTGAGTAGCTTTTTT

- **The silence sequence targeting the Piezo2 gene is as follows:**

Piezo2-sh1：GCACCTGATTGGACTTTATTTCAAGAGAATAAAGTCCAATCAGGTGCTTTTTT

Piezo2-sh2：GCAGAACCATTGTTAAGAATTCAAGAGATTCTTAACAATGGTTCTGCTTTTTT

Piezo2-sh3：GGACTTAGATGGAGAAGAATTCAAGAGATTCTTCTCCATCTAAGTCCTTTTTT

Piezo2-sh4：GCTGTGTACTTCTTTGTATTTCAAGAGAATACAAAGAAGTACACAGCTTTTTT

**Table 2. Abdominal withdrawal reflex (AWR) scoring criteria.**

| **Scores** | **Characteristic** |
| --- | --- |
| **Score 0** | No behavioral response to colorectal distension (CRD). |
| **Score 1** | Immobile during distension of colorectum (CR) and occasional appearance of brief head motion after a pause at the onset of the stimulation. |
| **Score 2** | A mild contraction of abdominal muscles, but no lifting of abdomen off the platform. |
| **Score 3** | A strong contraction of abdominal muscles and lifting of abdomen off the platform, no lifting of pelvic structure off the platform. |
| **Score 4** | Arching body and lifting of pelvic structure and scrotum. |

**Table 3. Method of primer design and primer sequences for quantitative real-time PCR**

Genes were searched based on the genome sequence of the mouse (Mus musculus (house mouse)) published on the NCBI website (<https://www.ncbi.nlm.nih.gov/>). Primer sequences were designed based on shared cDNA sequences of two variants of the *5HT_3_R* gene (Gene ID: 15561), or shared cDNA sequences of four variants of the *Piezo2* gene (Gene ID: 667742), respectively. Specific primers were designed with the Primer Premier 5.0 software by General Biol (Anhui) Co., Ltd.

| **Gene name** | **Gene ID** | **Forward** | **Reverse** |
| --- | --- | --- | --- |
| ***GAPDH*** | 14433 | 5’-CCTCGTCCCGTAGACAATG-3’ | 5’-TGAGGTCAATGAAGGGGTCGT-3’ |
| ***Epac1*** | 223864 | 5’-TACGGATGAAGAACTGGACCTGA-3’ | 5’-TTCCCGCTTCACCGAGTTAG-3’ |
| ***Piezo2*** | 667742 | 5’-AGTCTGGAAGCTCAACACCG-3’ | 5’-AGCCAGATGGTCAGACTTGC-3’ |
| ***5-HT_3_R*** | 15561 | 5’-GACCATCTTCATTGTGCGC-3’ | 5’-GGCTGAGCAGTCATCAGTCT-3’ |

**Table 4. Body weight of mice during the experiment Ⅱ.**

Data from body weight were analyzed using generalized estimating equations (GEE) due to the correlated structure of data from repeated measures at different time points.

^#^: model versus control, *P* < 0.05; ^&^: EA versus control, *P* < 0.05; ^△^: sham EA versus control, *P* < 0.05; ^**, ***^: in the same group, latter time-point versus former time-point, ^**^*P* < 0.01, ^***^*P* < 0.001

|  | **Control** | **Model** | **EA** | **Sham EA** |
| --- | --- | --- | --- | --- |
| **Day 0** | 21.03±0.46 | 20.45±0.29 | 21.14±0.32 | 21.21±0.27 |
| **Day 7** | 21.55±0.60 | 20.68±0.27 | 21.23±0.54 | 20.30±0.68 ^***^ |
| **Day 14** | 23.47±0.58^***^ | 22.00±0.47^***, #^ | 22.73±0.39^***, &^ | 22.31±0.49 ^***, △^ |
| **Day 21** | 24.60±0.59^***^ | 23.46±0.61^***^ | 24.35±0.44^***^ | 23.51±0.55 ^***^ |
| **Day 28** | 25.04±0.62^**^ | 24.18±0.65^***^ | 25.16±0.44^***^ | 24.48±0.45 ^***^ |
| **Day 36** | 25.56±0.62^***^ | 25.00±0.70^***^ | 26.17±0.42^***^ | 25.36±0.44^***^ |
